# A Dance Movement Psychotherapy Intervention for the Wellbeing of Children With an Autism Spectrum Disorder: A Pilot Intervention Study

**DOI:** 10.3389/fpsyg.2021.588418

**Published:** 2021-07-19

**Authors:** Supritha Aithal, Vicky Karkou, Stergios Makris, Themis Karaminis, Joanne Powell

**Affiliations:** ^1^Research Centre for Arts and Wellbeing, School of Applied Health and Social Care, Edge Hill University, Ormskirk, United Kingdom; ^2^Research Centre for Arts and Wellbeing, Department of Psychology, Edge Hill University, Ormskirk, United Kingdom

**Keywords:** Autism spectrum disorder, dance movement psychotherapy, wellbeing, pilot study, arts therapies, social and communication

## Abstract

**Background:**

Sustaining the wellbeing for children with an autism spectrum disorder (ASD) can be highly demanding. Dance Movement Psychotherapy (DMP), a form of psychotherapy with a non-verbal character, may present as a relevant intervention option for this group of children.

**Methods:**

A protocol-based group DMP intervention was developed and implemented in two special educational needs schools in the North West of England. We aimed to investigate the effects of DMP on children with ASD using the Social Communication Questionnaire (SCQ) and Strengths and Difficulties Questionnaire (SDQ). Twenty-six children aged between 8 and 13 years (mean age = 10.65 years) with ASD were randomly allocated to DMP and a control group with standard care, following a crossover research design.

**Results:**

Results showed no significant carryover or period effects for either the SCQ or SDQ (*p* > 0.05). A significant intervention effect was found only for SCQ (*p* = 0.005) but not for SDQ (*p* > 0.05). ANCOVAs were performed on the data before the crossover to test for differences in SCQ and SDQ scores between the DMP intervention and control groups while controlling for pre-intervention scores. Those in the DMP intervention group presented significantly lower SCQ scores following the intervention period than those in the control group (*p* = 0.001). No significant differences in post-intervention SDQ scores were found between DMP intervention and control groups (*p* = 0.2). However, minimal clinically important differences (MCID) were reached for both SCQ and SDQ measures before crossover for those in the DMP intervention group. Moreover, repeated measures ANOVAs performed on SCQ and SDQ measures following crossover were significant, with the change in both SCQ (*p* = 0.001) and SDQ (*p* = 0.009) pre-and post-intervention being significantly greater for those in the DMP intervention than the control group.

**Conclusion:**

The pilot DMP intervention has shown promising results on the social and emotional wellbeing of children with ASD irrespective of whether they preferred verbal or non-verbal mode of communication. Limitations and appropriateness of the research methods implemented in this study for their use in a large RCT are discussed in detail. Overall, our findings highlight the value of creative therapies for improving the lives of young vulnerable groups.

## Introduction

Autism spectrum disorder (ASD) refers to a group of complex and pervasive developmental conditions with unique strengths and challenges (American Psychiatric Association, 2013; Volkmar et al., 2014). The word “spectrum” in ASD highlights the heterogeneity in individuals with ASD; their abilities and challenges may fall anywhere in this spectrum. Their challenges particularly impact their social, communication, and behavioral patterns early in life and persist throughout the life time, while their strengths vary drastically and can be found, but are not limited, to areas such as memory, music, mathematics (Lord et al., 2018; National Institute for Mental Health (NIMH), 2020). With these abilities and challenges, children with ASD encounter several issues in their interactions with others and this might impact on their wellbeing.

Wellbeing is a broad and multidimensional notion, frequently linked to important societal factors such as mental health, happiness, productivity and health care costs (Kraatz et al., 2016). This study is grounded on the definition of wellbeing proposed by Dodge et al. (2012), which considers the principles of equilibrium/homeostasis and the fluctuating state between challenges and resources. Too many challenges, as well as excessive resources, are equally responsible for an imbalance. Therefore, wellbeing as a dynamic process with an attempt to strike a balance point between an individual’s resource pool and the challenges faced (Dodge et al., 2012).

Children with ASD may encounter numerous emotional, social and communication challenges in life. This could create an imbalance between resources and challenges in their wellbeing. To maintain a balanced state, children will have to adapt their resources and challenges accordingly. Challenges refer to difficult life events and resources are linked to situations where everyone develops relevant skills to cope with the challenges they face. In this study, we focus on two different dimensions of wellbeing of children with ASD as highlighted by the National Institute for Health and Care Excellence (National Institute for Health and Clinical Excellence (NICE), 2017): (i) emotional wellbeing, which includes being happy and confident and not anxious or depressed and (ii) social wellbeing, which considers factors such as effective communication, having good relationships with others and not having behavioral problems, that is, not being disruptive, violent or a bully.

Autism spectrum disorder has become one of the most common developmental disabilities in children and has been reported to be the most expensive condition in the United Kingdom (UK) (Buescher et al., 2014; National Autistic Society (NAS), 2020). In addition, in the same country, occurrence of ASD is at least four times more in males than in females and is present in all ethnic and socioeconomic groups. As per the Centers for Disease Control’s Autism and Developmental Disabilities Monitoring (ADDM) Network (Christensen et al., 2016) about 1 in 54 children has been identified with ASD in 2016 while it was 1 in 150 in the year 2000. The high rate of ASD shows mounting demands for better intervention options and provision of support for children with ASD. This fact led the sixty-seventh World Health Assembly, supported by more than 60 countries, to undertake a resolution for “comprehensive and coordinated efforts for the management of ASD” (World Health Organization (WHO), 2014).

The World Health Organization (WHO) (2018) recognizes the complex health care needs and inadequate services and support available for people with ASD. Amongst several approaches promising intervention results, there are only a handful of them which have demonstrated evidence of their effectiveness for children and adolescents with ASD (Simpson, 2005). The National Autistic Society (NAS) (2020) recognizes a range of approaches which can be broadly classified under those that are communication-based, behavioral and educational. Although these evidence-based approaches have data to support their usefulness, many clinicians and researchers raise concerns about their attitudes, goals and methods (e.g., Silberman, 2015; Baron-Cohen, 2017; Mottron, 2017). The most common criticisms of the approaches are that they are addressing only the problems faced by children with ASD, ignoring the possibility of also focusing on their strengths (Mottron, 2017). Further criticisms are that these interventions encourage socially appropriate behaviors which are normative and norm-centric by oppressing children with ASD and attempting to change their core characteristics. Nind (1999) for example, argues that these interventions do not allow children to *express* who they really are, and they fail to promote happiness and the unique strengths possessed by people with ASD. Furthermore, National Institute for Health and Care Excellence (NICE) (2020) recommends Cognitive Behavioral Therapy (CBT) and emotion recognition training for co-existing mental health issues faced by children with ASD. However, these interventions are only suitable for children with appropriate verbal and cognitive capacity to engage in such interventions. It is challenging to reach children at their own level to communicate effectively and to promote their strengths. It is argued that arts therapies, including dance, music, art and drama, have the potential to penetrate through the barrier of verbal communication by incorporating non-directive, creative and predominantly non-verbal strategies to support the wellbeing and growth of children and young people (Moula et al., 2020). The findings of a systematic review that synthesized outcomes from children’s perspective on attending arts therapies highlighted that children found arts therapies to be useful in improving their socio-communication abilities and emotional wellbeing (Moula et al., 2020). Children also reported that arts therapies helped them to build their resilience, academic learning, and manage aggressive behavior.

### DMP for Children With ASD

Dance movement psychotherapy (DMP) is one of the arts therapies and works with the principles of initiating the process of *bringing change* for both therapists and individuals with ASD (Scharoun et al., 2014). This is unlike many other interventions where the change is expected only from individuals with ASD. Earlier DMP interventions for children with ASD fostered children’s creative expression and showed an appreciation of both their strengths and weaknesses (Adler, 1968; Siegel, 1973; Erfer, 1995). Using mirroring as one of the key techniques, therapists and clients were supported to experience each other’s viewpoint empathetically and thus enable mutual understanding and change for both clients and therapists.

Mirroring is an important tool used by dance movement psychotherapists to the current date. It often involves an affective attunement to the non-verbal presentation and movement preferences of the children (Meekums, 2002). Stern (2000) argues that these attuned processes facilitate integration and organization of sensory experiences and self-regulation. These are crucial for the development of intersubjectivity which can further social communication in children with ASD. The outcomes of DMP interventions are thus assumed to be changes imbibed out of self-will or autonomy as opposed to changes that are imposed. In this respect, DMP is fundamentally distinct from other available interventions for children with ASD while it is can be applied in a wide range of settings including, for instance, special educational needs (SEN) schools, home, hospital, and community settings.

Research in DMP for children with ASD is still limited. Early DMP practitioners have highlighted the potential of DMP to offer better mental health, more independence and creative opportunities for early interventions for children with ASD (Adler, 1968; Siegel, 1973; Kalish, 1977; Erfer, 1995; Loman, 1995; Parteli, 1995; Scharoun et al., 2014). These have been largely documented using case studies and clinical reports. In the past decade, studies with a clearer focus on research are being conducted. The systematic literature review completed for DMP practice with children below 16 years (Aithal, 2020) synthesized findings from seven studies with clear research designs. The review included two quantitative studies (Hartshorn et al., 2001; Chiang et al., 2016), three qualitative (Wengrower, 2010; Houghton and Beebe, 2016; Devereaux, 2017), one artistic inquiry (Athanasiadou and Karkou, 2017) and one mixed-methods thesis (Samaritter, 2015). It concluded that DMP can potentially promote the social, physical, emotional as well as cognitive wellbeing of children with ASD. However, evidence for its effectiveness still remained inconclusive as many studies made claims about the effectiveness of the intervention without substantial supporting evidence. In addition, most of the studies reviewed were identified with issues in the quality of the research design, execution of the methods and reporting of the results.

Similarly, Marchant et al. (2018) conducted another systematic review on DMP for adults with ASD. This review also considered seven studies; out of them most also were quantitative (Mateos-Moreno and Atencia-Doña, 2013; Koch et al., 2015; Hildebrandt et al., 2016; Koehne et al., 2016; Manders, 2016), while only two were qualitative studies (Wadsworth and Hackett, 2014; Edwards, 2015). The largest study among them was Hildebrandt et al. (2016) with 78 participants and the study found symptom reduction on the overall negative symptoms in ASD was greater in the intervention group. However, in this randomized controlled trial, the effect size was only significant at 0.10 level and there was a high amount of data attrition. A study of this type and size for children with ASD was not available. Furthermore, in both systematic reviews, the studies were too diverse to be eligible for conducting meta-analysis and for the calculation of the collective effect size (Marchant et al., 2018; Aithal, 2020). Thus, despite several decades of DMP work with persons on the autism spectrum the effectiveness of DMP still remains inconclusive.

So far, randomized controlled trials have been conducted with adults on the autism spectrum only. DMP studies with children on the autism spectrum have not been able to provide clear information on dosage (frequency and duration of DMP sessions), theoretical frameworks, therapeutic techniques and establish the effectiveness of the intervention. Therefore, well-designed studies on the impact of DMP for children with ASD are warranted.

### Overall Aim, Research Questions and Hypothesis

This DMP intervention pilot study adopted a crossover research design with a primary intention to investigate the existence of an intervention effect and explore the contribution of DMP toward the wellbeing of children with ASD. In particular, the focus was on changes in emotional and social wellbeing of children with ASD as a result of DMP intervention in comparison with their standard care routine. As a pilot study, it was designed to inform the development of a larger randomized controlled trial with children with ASD and did not require large sample size and statistical power (Thabane et al., 2010).

The primary research question was therefore:

(1) What are the effects of DMP on social and emotional wellbeing of children with ASD?

We also asked additional questions, one of which is answered in this paper:

(2) What is the appropriateness of the research methods adopted in this study for their use in a large RCT?

The study explored if DMP can enhance the resources or strengths of children with ASD by developing relevant skills to cope with the challenges. Hence, the reduction in the challenges or difficulties indicated a positive impact of the intervention. On the basis of this understanding of wellbeing, the following null hypothesis was used:

*H*_0 =_ DMP had no effect on measures of social and emotional wellbeing of children with ASD who participated in the intervention programme.

As this was a pilot study, our measure for determining if the DMP intervention was influencing our outcome measures was whether or not our change score reached our predetermined minimal clinically important difference (MCID). The MCID is often used in clinical intervention studies that adopt patient reported outcome measures. The approach allows us to conclude whether an intervention is having a clinically significant effect irrespective of the statistical significance value obtained (Jaeschke et al., 1989; Cook, 2008).

## Materials and Methods

This article focused on the quantitative strand of a larger mixed-methods pilot DMP intervention based on a crossover and convergent research design (Creswell, 2018). Qualitative and arts-based findings from the broader study are not presented for reasons of space. This intervention study was block randomized, controlled and engaged in finding the effects of the protocol-based DMP on social, communication, emotional and behavioral aspects of ASD. The study was approved by the ethics committee of the Faculty of Arts and Sciences at Edge Hill University.

### Participants

Participants were recruited from two special educational needs (SEN) schools in the North West of England. Children below 16 years with a diagnosis of ASD as per the Diagnostic and Statistical Manual, Fifth Edition (DSM-5), children’s assent, willingness and parent’s consent for them to participate were the eligibility criteria for participation in the study. Children with a diverse severity of ASD, language, cognitive and physical abilities and associated problems were included.

### Severity and Mode of Expression

Children’s mode of expression was categorized as verbal or non-verbal. The preferred mode of expression was categorized as “verbal” if the difference between the chronological age of children and expressive language age was less than 2 years. Such children (8–13 years) were able to express using sentences with intact syntax, semantics, phonology and major issues with just the pragmatic component of language. All those children who used only some content words or no words, predominantly used Makaton (Brownjohn, 1988), and the difference between their verbal expressive language and their chronological age was greater than 2 years came under the “non-verbal” category as their preferred mode. Participants’ difficulties were also categorized according to ASD severity based on direct observation using Childhood Autism Rating Scale Second Edition CARS2 (Schopler et al., 2010). The researcher subjectively rated participants’ difficulties on the fifteen items of CARS as follows: (i) mild – if they required support and faced difficulties in social situations only; (ii) moderate – if the children required substantial support; and (iii) severe when children required very substantial support to carry out basic daily living tasks.

### Study Design

The research studied the effects of the proposed DMP intervention by conducting a pilot intervention with a crossover design (Jones and Kenward, 2014). The Crossover design comprised the factors **Group** (DMP versus standard care group) and **Time** (before versus after the intervention) at two time **Periods** before crossover (period 1) and after crossover (period 2). The end and start of these periods were separated by 1 month. A crossover design was chosen because the approach can accommodate low sample sizes, while in terms of research ethics, it provides intervention opportunities to all the participants. The crossover design was planned with equal rigor as in a parallel-group trial to make sure that the crossover approach meets the criteria in terms of type I and type II error risks (Wellek and Blettner, 2012). Finally, the crossover design allowed for comparisons of DMP and standard care within the same participants and reduced within-subject variability (Jones and Kenward, 2014).

### Randomization

Block randomization (Altman and Bland, 1999), a technique that helps to randomize the participants into clusters to receive intervention (A then B or B then A) was used. Blocks were defined as small, predetermined cluster assignments, which help keeping the numbers of participants in each group similar and create a balance (Frane, 1998). Participants were manually divided using a shuffled deck of sealed cards to decide the order of intervention. When parents picked a card with an even number, children were allocated into the DMP intervention group in period 1; when they picked an odd number children were allocated to the control condition where they maintained their standard routine as usual in period 1. Two clusters of the participants received DMP (A) followed by standard care (B) while the other three clusters received standard care followed by DMP (A then B/B then A design). The groups were unchanged once the participants were allocated to groups. Blinding of the participants and the researchers was not feasible as parents and teachers who filled out the questionnaires and children who participated in the sessions were aware of the intervention phase. Placebo group sessions were not provided due to logistical constraints and limited resources. The researcher was involved during DMP sessions to gather qualitative and arts-based data (for the qualitative strand of the broader study).

### Outcome Measures

The outcome measures were the Strengths and difficulties Questionnaire (SDQ) developed by Goodman (1999) and the Social communication Questionnaire (SCQ) developed by Rutter et al. (2003). Both measures have been found helpful in intervention planning, educational intervention and measurement of change over time following an intervention (Mieloo et al., 2012; Avcil et al., 2015; Stone et al., 2015). This makes them more appropriate than other measures such as the ADOS which is not meant to detect intervention effects (Goodman et al., 2000; Bieleninik et al., 2017).

The SDQ is a behavioral assessment tool used to gain inputs on child’s emotional and social wellbeing from the teacher’s perspective. It consists of 25 items divided between five scales: emotional symptoms, conduct problems, hyperactivity/inattention, peer relationship problems and prosocial behavior. Responses are given to each item on a 3-point scale (Not True, Somewhat True and True). Although the questionnaire allows separate calculation for each subscale, it also provides an overall score using raw scores from the first four subscales, with a higher score indicating a greater degree of difficulty. A large national survey of child and adolescent mental health carried out by the Office for National Statistics in Britain found the average SDQ scores for a 5–15 year old sample (forms completed by teachers) was Mean = 6.6, SD = 6.0 (Meltzer et al., 2003). The class teachers of the children answered the questionnaire before and after both period 1 and 2.

The SCQ is a psychological questionnaire designed to identify social and communication abilities of children from parents’ perspective. The *current version* of the form was used in the study as this provides an assessment of the child’s behavior over a recent period of time. The test consists of 40 yes/no questions appropriate for both verbal and non-verbal children, with higher scores indicating a greater number of social and communication difficulties. SCQ scores above the cut-off point of 15 indicates ASD and deviant social and communication patterns in them (Berument et al., 1999). Wiggins et al. (2007) found that the sensitivity and specificity of the SCQ were maximized at lower cut offs of twelve when used with younger children using non-verbal communication. Parents were invited to answer the questionnaire during the parent-teacher meeting before and after each term.

### Procedure

**FIGURE 1:**
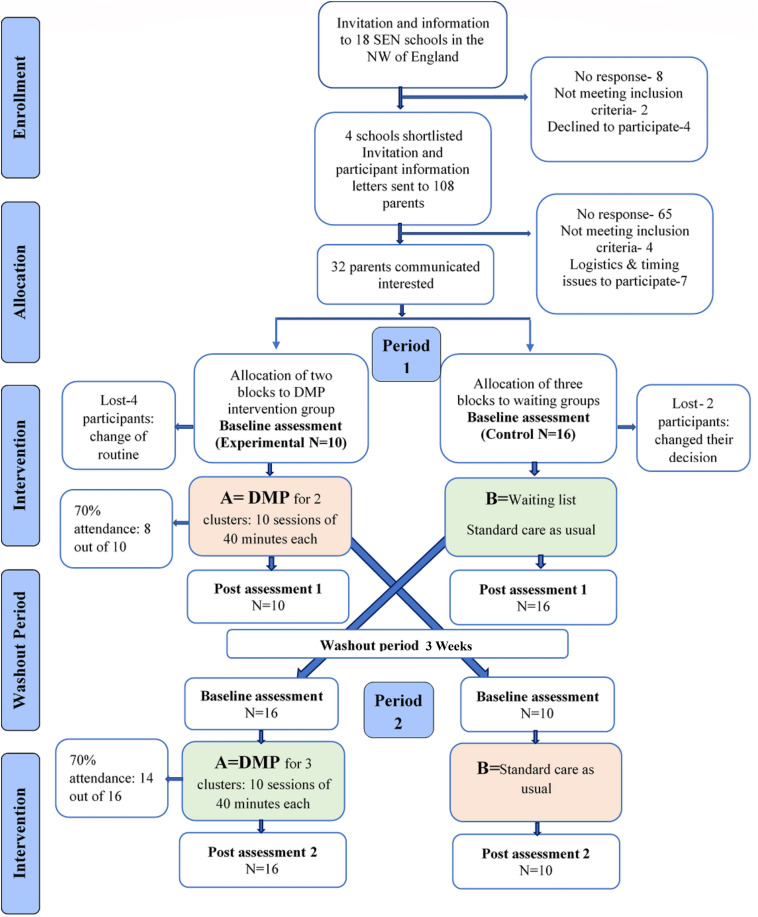
CONSORT flow diagram for each stage of the trial.

To report the progress through the phases of this study we have implemented the Consolidated Standards of Reporting Trials (CONSORT) guidelines with some slight modifications adapted to report a crossover randomized controlled trial. As shown in the CONSORT flow diagram (Figure 1) invitations were sent to 18 SEN schools. Following school visits only four schools were identified as suitable, based on the number of children available and responses from the parents. Two schools proceeded further to send out study documents i.e., participant information sheet, invitation letters and reply slips to 108 parents of children with ASD. A total of 32 parents communicated interest from both locations. Following participant drop-out, before the assessment (shown in Figure 1) 26 children were then grouped into five clusters and two clusters received the intervention in the first period while the other three clusters were in the waiting list (control group). In the second period of the trial the clusters were swapped from waiting group to intervention groups. Baseline and post assessments were carried out at both periods. Incorporating the DMP intervention into the school schedule enabled the current study to reduce or even eliminate missing data, as well as increase attendance at the DMP intervention sessions. In the current study 23/26 children attended 70% or more sessions, while the remaining 3/26 participants did not complete the intervention either because they did not accept the intervention, or they found it difficult to adjust to the change in their routine. There was only one participant who dropped out clearly stating that he liked science experiments more than dancing. Some children missed out sessions in between due to frequently falling sick, class trips, priority to certain school events and unavailability of the teaching assistants to escort the children with high dependency needs to the sessions.

Effectively, all outcome measures were obtained from all 26 participants and data from all 26 participants was included in the statistical analysis. Thus, the inclusion of data in the final analysis utilises an intention to treat (ITT) approach.

### Intervention

The intervention programme was developed based on the synthesized findings from the systematic literature review of DMP for children with ASD that was conducted as part of the first author’s doctoral study. The DMP intervention protocol was designed for ten sessions divided across four modules with a frequency of two sessions of 40 min every week. The sessions were built on an eclectic/integrative DMP framework (Karkou and Sanderson, 2006) that acknowledged useful ingredients from the reviewed literature (Aithal, 2020). As described in the Table 1, the intervention protocol comprised of eight principles which acted as the backbone to the practical application of the session objectives.

**TABLE 1:** Principles, modules and objectives of the ten DMP Intervention sessions.

**Principles**	
Focusing on therapeutic relationships Approaching participants with warmth Adopting an empathetic attitude Working with participants from where they are Working with existing strengths Considering attachment patterns Supporting sensory motor development Focusing on enhancing social skills	
**Modules**	**Session objectives**

Getting familiar with the process, each other and space	To provide an overall introduction to the process and introduce to each other To explore the space
Focusing on self, props and dyads	To identify personal strengths To find new ways of moving together with props To move in new ways with self and in pairs
Supporting group work	To engage in group work with sensory motor explorations To engage in group work with symbolic and imaginative play To engage in group work with embodied role play
Closing the process	To concretize important aspects of the therapeutic journey To bring the process To a suitable closure

Each session began with a consistent opening ritual where children could express their readiness to take part in the sessions. It was then followed by a playful warm up section and further developed to meet the objectives of the sessions. The themes formed the core part of the sessions and they varied across the clusters depending on the needs of the participants in the cluster. The sessions were gradually brought to closure by cooling down and using a closing ritual. A wide range of techniques including mirroring, sensorimotor explorations creatively merged alongside the use of play techniques, rhythm and props. These were used in solo, dyad and group configurations.

Sessions were structured around the strengths of the participants where the therapist initiated the movements following the child’s lead to work on embodied movement experiences. The therapist’s movement patterns resonated with their interests and energy levels (Mottron, 2017). For example, in session three, children explored embodying the movement qualities of their favourite animals. They took turns to display various movement qualities such as the lightness of a bird or the power of a lion in these roles. Each individual made unique contributions to the group. For example, one child brought in gentle movement qualities to improvise while another child used swift and sharp movement qualities. A child in a wheelchair was encouraged to move and make his own sounds through clapping, body tapping and vocalizations that the group used to move to. The complete design of the protocol along with the conceptual background and the findings of the fidelity assessment have been detailed as a separate paper (Aithal et al., in preparation).

A qualified and ADMP UK registered therapist facilitated all five DMP clusters with children at two different locations. The therapist was a female DMP practitioner and a sports psychologist with more than 5 years of clinical experience. The sessions were clinically supervised by the director of this doctoral study.

### Statistical Analysis and Measurements

**FIGURE 2:**
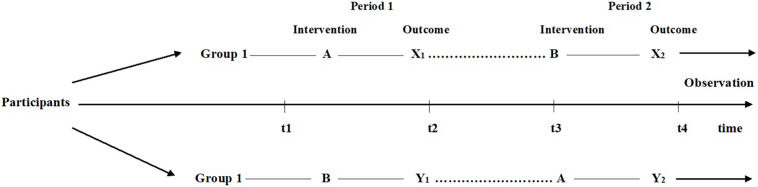
Crossover design to test carry over, intervention and period effects. A = Dance Movement Psychotherapy (DMP); B = Standard Care; X_1,_ X_2,_ Y_1,_ Y_2_ = Post Intervention Measurement Timepoints. (Figure adapted from Wellek and Blettner, 2012).

All data from the 26 participants who completed the study were analyzed using the Statistical Package for the Social Sciences version 25 for Windows (SPSS Inc., Chicago, IL, United States). Figure 2 illustrates the crossover design as it was applied in the current study. Based on this design we analyzed the following measures.

#### Baseline Group Differences

As all conditions were met for conducting the parametric tests, a one-way ANOVA was performed to test for significant differences in pre-intervention scores for SDQ and SCQ at t1 for Group 1 and Group 2. This was to determine whether there were any differences in baseline scores between the two groups before receiving either intervention.

#### Group Differences Before Crossover

Two separate ANCOVAs were performed on the data from period 1 (i.e., before crossover) to test for differences in SCQ and SDQ scores between those receiving the DMP intervention and the control group, following the intervention period, while controlling for pre-intervention scores. The participants mode of expression (categorized as verbal and non-verbal) was entered into the model as well as the interaction term between group (DMP intervention/control) and mode of expression (verbal/non-verbal).

#### Intervention Effects

We calculated intervention effects were calculated for the crossover design using the approach outlined by Jones and Kenward (2003). Specifically, we calculated carry-over effects as well as intervention and period effects for SDQ and SCQ scores.

As shown in Figure 2, group 1 consisted of children who received intervention A (DMP intervention) first followed by intervention B (standard care) [i.e., A then B]. Group 2 involved children who received intervention B first followed by intervention A [i.e., B then A]. All measures observed in the crossover analysis i.e., X_1_, X_2_, Y_1_, and Y_2_ were post-intervention scores and the timepoints indicated the four points in time at which each group was assessed for all the outcome measures in the study. Carryover effects were calculated by comparing the sum of values over both intervention periods between group 1 (ΣX_1_ + ΣX_2_) and group 2 (ΣY_1_ + ΣY_2_) using a 2-sample *t*-test. The intervention effect was tested for by comparing the difference between intervention A and intervention B for the 2 groups. For group 1 the differences were calculated for after time period 1 minus after time period 2 (i.e., X_1_ – X_2_), while for group 2 the differences were for after time period 2 minus those after time period 1 (i.e., Y_2_ – Y_1_).

The period effect measured whether there was a change over time irrespective of intervention. Period effects were calculated by comparing the difference between period 1 and period 2 for the 2 groups. For group 1 the difference was calculated as X_1_ – X_2_ and for group 2 the difference was calculated as Y_1_ – Y_2_.

Two repeated-measures ANOVAs were performed on the total data set, following crossover to test whether the change in SCQ and SDQ scores differed between DMP intervention and control groups. The change in pre-and post-intervention measures was calculated for the DMP intervention and control conditions which were used as the two dependent variables in the model, classified as the within-subjects factor, intervention. A between-subjects factor was entered for group (group 1/group 2) which specifies the order in which participants received each condition (i.e., A then B versus B then A). This analysis was performed to test whether the change in pre-and post-intervention scores differed between interventions received while controlling for the crossover design feature of the study. To calculate the change score equivalent to the Minimal Clinically Important Difference (MCID), the standard deviation of the baseline scores was multiplied by 0.2 (the small effect size) (Samsa et al., 1999). MCID was calculated for SCQ and SDQ *before* crossover. Eta squared was also calculated to determine effect size for all tests performed and then converted to Cohen’s *d* using free online software by Psychometrica (Lenhard and Lenhard, 2017). An alpha level was used to set the standard and a level of 0.05 was chosen for determining statistical significance. An effect size between 0.2 and 0.4 was considered as “small effect” and an effect size > 0.8 was deemed as a large effect (Lenhard and Lenhard, 2017).

## Results

The total sample considered in the statistical analysis included 26 children (21 males) aged between 8 and 13 years from two different centers (see Table 2). All 26 participants were British citizens with 19/26 being white. Most (24/26) spoke English as their first language, with the remaining two being native Spanish speakers. A 16 of them preferred verbal communication while the remaining 10 communicated using alternative non-verbal modes. As per the researcher’s ratings on the CARS2 (Schopler et al., 2010) measure at the baseline evaluation, 7/26 were classed as having mild difficulties, 12/26 as having moderate, and 7/26 as severe difficulties across social, emotional, communication, and cognitive aspects.

**TABLE 2:** Baseline characteristics.

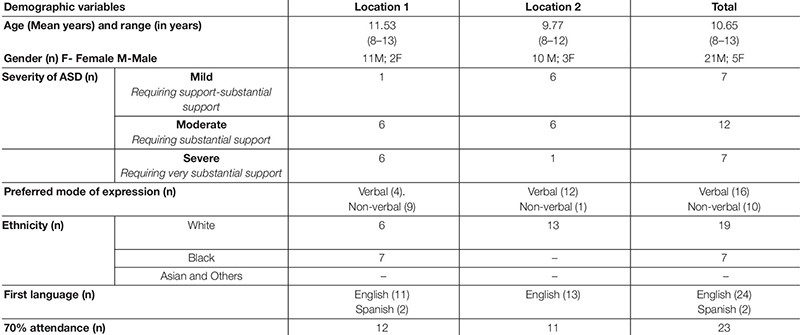

Participants had no previous experience with DMP, but they had taken part in arts sessions as part of their school activities. They were not familiar with either the therapist or the researcher who was the co-facilitator before the start of the sessions. Teaching assistants took part along with children. The number of teaching assistants and people varied according to their availability, school’s schedule and participants’ needs and hence was an extraneous variable which went uncontrolled and unmeasured.

Means and standard deviation for SCQ and SDQ scores pre-and post-intervention before and following crossover, separated by DMP intervention and control groups is shown in Table 3.

**TABLE 3:** Means and standard deviations (bracketed) for SCQ and SDQ scores pre- and post-intervention separated by group and intervention as well as the total.

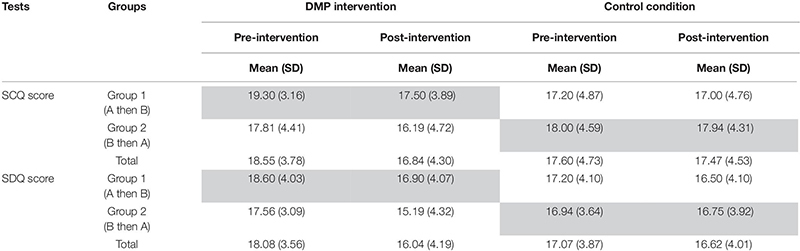

*Note that scores shaded grey represent the data set before crossover was applied. SCQ, social communication questionnaire; SDQ, Strengths and difficulties questionnaire; SD, standard deviation; A, Dance Movement Psychotherapy Intervention; B, Standard Care.*

Across all groups average SCQ scores are higher than the SCQ cut-off score (i.e., mean SCQ > 15). Similarly mean scores for the total SDQ score are three times higher (i.e., 18.31 at pre-intervention across all participants) than the normative data of typically developing children in Britain i.e., mean = 6.6, SD = 6.0 (Meltzer et al., 2003). Results from the one-way ANOVA revealed no significant difference in either SCQ or SDQ score at pre-intervention between DMP intervention and control groups (*p* > 0.05).

Results for the 2-sample *t*-tests performed to determine significant carryover, intervention and period effects for the crossover design showed no significant carryover or period effects for either the SCQ or SDQ (*p* > 0.05). A significant intervention effect was found for SCQ (*t*_24_ = 3.067, *p* = 0.005). The mean difference in intervention effect was 2.25 (95%CI: 0.74, 3.76). No significant intervention effect was found for SDQ; however, this was close to the boundary of significance (*t*_24_ = 1.895, *p* = 0.07). The mean difference in intervention effect for SDQ was 1.96 (95% CI: −0.17, 4.10).

Findings from the ANCOVAs showed significant differences in SCQ between DMP intervention and control groups before crossover was applied (*F*_1_,_21_ = 15.715, *p* = 0.001, Cohen’s *d* = 0.09). Specifically, those in the DMP intervention group presented lower SCQ scores following the intervention period than those in the control group (standard care). Results showed no significant difference in post-intervention SDQ scores between DMP intervention and control groups before the crossover design (*F*_1_,_21_ = 1.853, *p* = 0.2, Cohen’s *d* = 0.02). The mode of expression and the interaction term group^∗^mode of expression were not significant in the ANCOVA model for either SCQ or SDQ (*p* > 0.05).

Furthermore, results from the ANCOVAs showed that the DMP intervention was having a significantly greater effect than standard care on SCQ scores but not on SDQ scores. However, the MCIDs indicate that the intervention was achieving the minimal clinically important difference, as assessed between pre- and post-measures, for both SCQ and SDQ outcomes for those taking part in the DMP intervention but not the standard care (control group). MCIDs were calculated for SCQ and SDQ scores *before* crossover. Results showed that for SCQ the mean difference between pre- and post-intervention score was greater than the MCID (0.816) for the DMP intervention group (i.e., 1.8 > 0.816), but not for the control group (i.e., 0.06 < 0.816). Similar results were found for SDQ scores with the mean difference in pre- and post-intervention SDQ score being greater than the MCID (0.762) for the DMP intervention (i.e., 1.7 > 0.762) but not the control condition (i.e., 0.19 < 0.762). These results suggest that whilst the difference in post-intervention scores for SCQ and SDQ between DMP intervention and control groups was not significant, the mean difference in pre- and post-intervention scores for the DMP intervention but not the control condition achieved the MCID for a small effect size.

**FIGURE 3:**
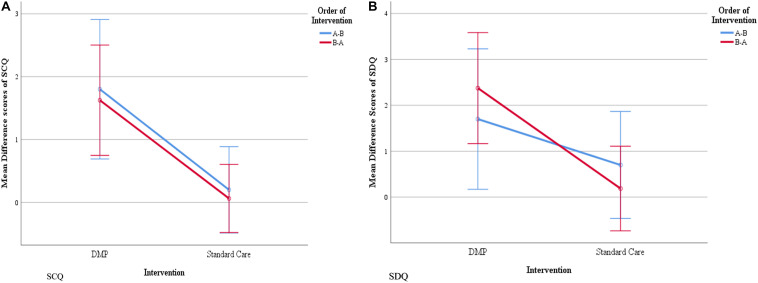
Mean difference in pre- and post- intervention scores for SCQ **(A)** and SDQ **(B)**, separated by the order of interventions received. The blue line represents the order of interventions **(A)** (DMP intervention) then **(B)** (standard care) and the red line represents the order of intervention **(B)** then **(A)**. Blue and red vertical lines, parallel to *Y* axis represent error bars with 95% confidence intervals.

Results from the repeated measures ANOVAs which were performed on the total data set *after* crossover show that the change in pre- and post-intervention SCQ scores differed significantly between DMP intervention and control groups (*F*_1,24_ = 13.891, *p* = 0.001, Cohen’s *d* = 1.523). Those in the DMP intervention group showed a significantly greater change in SCQ scores than those receiving standard care (i.e., 1.69 versus 0.12, respectively). The effect of the intervention on change in scores did not differ between the order (i.e., A then B/B then A) in which the interventions were received (*p* > 0.05). Results also showed that the change in pre- and post-intervention SDQ scores differed between interventions (*F*_1_,_24_ = 7.963, *p* = 0.009, Cohen’s *d* = 1.127); specifically those in the DMP intervention group showed a significantly greater change in SDQ score than those receiving the standard care (i.e., 2.12 versus 0.38, respectively). The effect of intervention condition on the change in SDQ score did not differ between the order in which interventions were received (*p* > 0.05). Figures 3A,B present line graphs showing the mean difference in pre- and post-intervention scores for SCQ (Figure 3A) and for SDQ (Figure 3B), separated by the order of interventions received.

## Discussion

### Effects of DMP on Social and Emotional Wellbeing of Children With ASD

This section discusses the first research question “what are the effects of DMP on social and emotional wellbeing of children with ASD?” As indicated in the literature, the strengths and struggles of children with ASD are unique (Mottron, 2017) and this DMP-based study was designed to work with existing strengths to promote their wellbeing. This study was piloted to evaluate the impact of an DMP intervention protocol based on the findings of a systematic review (Aithal, 2020) on social, communication and overall psychological wellbeing of children with ASD after ten DMP sessions using a crossover research design. Minimal clinically important differences (MCID) were achieved *before* crossover for both outcome measures used in the study i.e., social communication questionnaire (SCQ) and strengths and difficulties questionnaire (SDQ) in the DMP intervention group. Differences in post-intervention measures for SCQ but not SDQ were significantly higher for those in the DMP intervention compared to the control group. Following the application of the crossover design, however, results did show that the change in SCQ and SDQ scores from pre- to post-intervention were significantly greater in the DMP intervention group than controls. Overall, the results show that the DMP intervention was having a clinically and statistically significant effect on the key outcome measures used in the study with stronger effects on social and communication aspects.

Findings reveal that from a parents’ perspective (assessed using the Social Communication Questionnaire; SCQ) children have gained from the short-term DMP intervention on social and communication abilities, when controlling for the child’s preferred mode of communication. Similarly, improvements were observed from the teacher’s perspective, as assessed by the SDQ, which is a measure of emotional, conduct, hyperactivity, peer relationships and pro-social behavior also when controlling for the child’s preferred mode of communication. This reflects that regardless of the heterogenous spectrum of abilities presented by children with ASD, DMP has been successful to further social and communication aspects of the children within a short period. This is likely because the non-verbal elements promoted in DMP cannot only enhance non-verbal expressions but can facilitate verbal communication as well. The results are coherent with the notion that the creative arts, in general, encourage social communication, which may otherwise be impeded by sensory, motor and social difficulties (Sharda et al., 2018).

Theoretically, DMP for individuals with ASD have been extensively informed from theories of intersubjectivity and embodied simulation theories (Hildebrandt et al., 2016). Trevarthen and Delafield-Butt (2013) argue that variations in body movements, motor control and processing sensorimotor information influence affective engagement and emotional regulation. A dysregulation of emotional perception and expression may often manifest as peculiar behaviors, anger, aggression, depression. Anxiety, self-stimulation or even as self-injurious behaviors (Mazefsky and White, 2014). Subsequently, people in general find it difficult to understand and respond to their exaggerated or understated expressions. Thus, emotional dysregulation can turn into a barrier for relationships and communication between individuals with ASD and others. It might be possible that DMP could have provided opportunities for children to engage affectively in these sessions enabling emotional regulation and opportunities to communicate with others. These findings are in line with the outputs of the earlier studies included in the systematic review (Samaritter, 2015; Chiang et al., 2016; Athanasiadou and Karkou, 2017; Devereaux, 2017).

Another reason that may explain identified improvements in social and emotional aspects of children is that the movements encouraged in the DMP intervention protocol were initiated following the child’s lead and resonated with the interests and energy levels of the children. These child-centered principles could have probably encouraged the participants to move ahead in the stages of intersubjectivity toward building or initiating social relations on their own, connect with others and express themselves. As Holt(1982, p145) states “we can give other people names and lists, but we cannot give them our mental structures; they must build their own.” This statement indicates the philosophical differences between this intervention and other evidence-based behavioral interventions mentioned earlier in this article that are used for children with ASD. In humanistic principles-informed arts therapies the idea that children take ownership of their own “change” is championed (Karkou and Sanderson, 2006). It is possible for children to eventually learn and articulate their actual emotions through behavioral approaches. However, clinical observation also suggests that when children are taught to name their emotion, the words and their body presentation may not be at synch. When children are posed with a simple question: “how are you?,” they may appear tensed, with a shrunken body and answer “I am happy.” This paradoxical verbal and non-verbal response could be because children are often reinforced by their environment to provide that answer. Subsequently, they *learn* that happy is the right and socially acceptable answer and may limit meaningful interactions. However, DMP which considers integration of mind and body as one of the foundational principles of practice may open new ways for meaningful and coherent interactions in children with ASD [Association for Dance Movement Psychotherapy (ADMP UK), 2013].

Overall, promising improvements in the SCQ and SCQ scores show that DMP might be a useful tool to facilitate the experience of “a state of wellbeing” (World Health Organization (WHO), 2001). It is possible, however, that a state of wellbeing refers to experiences that are transient. Henceforth, DMP could be an intervention offered to children with ASD as a way of being resourced to deal with challenges in emotional expression or communication. The study findings suggest that the DMP intervention was successful in catering for heterogenous abilities and needs. However, it is likely that not all the participants reached or experienced the state of wellbeing. Nevertheless, all of them have taken at least a step to get close to experience that state.

### Methodological Limitations and Future Directions

This section discusses the second research question “what is the appropriateness of the research methods adapted in this study for their use in a large RCT?” With regards to age, the demographics of the sample demonstrates that the age range (8–13) was more focused without much variance within the sample reflecting on no statistically significant difference between intervention and control groups at the baseline in period 1. The intervention protocol developed for this study emerged from the systematic review with a sample of an average age of 9.6 years from seven studies (Aithal, 2020). This review enriched the suitability of the therapeutic objectives of the intervention to the needs of present sample’s age range. According to Williams et al. (2008), the UK median age of diagnosis of ASD is around 4.5 years. Also, the challenges faced by adolescent groups are different from children due to the transitory phase to adulthood (Parsi and Elster, 2015). These facts could explain why research studies with children with ASD tend to include the 8–12-year olds. Hence, age of the target population can become an *ad hoc* factor in future studies to explore the differences in DMP approach as an early intervention and for adolescents separately by tailoring it to the needs of the age groups.

Most of the children who participated in this study were boys and it was not surprising as Loomes et al. (2017) reported that male and female ratio of 3:1 in the prevalence of ASD. Despite existing gender stereotypes and stigma associated with men in dance (Holdsworth, 2013), the current study did not encounter resistance from boys who participated in the study, except for one out of 21 boys who was vocal about not wanting to dance. This might be associated with better acceptance of DMP by the participants because of proliferation in the shift of gender stereotyped perception of dance among boys in recent years or children with ASD were not really influenced by the popular socio-cultural pressures (Reinders, 2013; Teixeira-Machado, 2015; Ferreira et al., 2019).

The sample included a heterogenous group of participants in terms of severity of ASD who were both verbal and non-verbal. The sample was scattered across mild moderate and severe categories but with a small number in each category to be included in the statistical analysis as a co-variate. It is still unclear if the severity of ASD would influence the effects of DMP on wellbeing. To take account of this spectrum, further explorations with larger sample size are needed. As mode of expression was included in analysis, the study argues that irrespective of the preferred mode of expression by children, short-term DMP intervention has displayed positive results in children with ASD. This highlights the strengths of arts therapies to sidestep the exacerbating impact of mode of communication in traditional talking psychological therapies. Researchers have noticed that school-age children with ASD often remain unengaged in social settings as there are limited opportunities for socio-communicative and emotional development despite regular interventions that children with ASD receive (Sharda et al., 2018). However, the results of the current study indicate that the use of alternate, creative and arts-based means of expression to support social communication enhance the prospect of developing meaningful relationships (Nathan, 2019).

The present study implemented a group approach to DMP considering the low-cost factor associated with group therapy and more opportunities for socialization in a group setting. In the past, there has been a mixed trend in DMP for children with ASD as the systematic review (Aithal, 2020) identified three studies with group therapy, three studies with individual therapy and one study with parent-child dyad. It is still unclear which configuration of DMP would be more helpful in relation to the profiles of the children, time and cost effectiveness.

It is worth contemplating if higher intensity, frequency and longer duration of the intervention would have an impact on the findings for several reasons. In evidence-based approaches such as ABA, Linstead et al. (2017), found that intensive ABA intervention yielded large, positive effects on language-related outcomes and moderate, positive effects on non-verbal IQ, social functioning, and daily living skills in children with ASD. In DMP studies involving participants with ASD in groups, the intervention was offered over one and a half to 2 months and sessions ranging from 30 min (Hartshorn et al., 2001; Houghton and Beebe, 2016; Devereaux, 2017) to 60 min (Chiang et al., 2016). These sessions were delivered once or twice a week totaling between eight (Athanasiadou and Karkou, 2017) to 20 sessions (Chiang et al., 2016). The current study considered ten sessions of 40 min to fit well within the school’s term time. Intervention effects as measured on SDQ, although showed a change close to the borderline to be statistically significant, possibly a greater number of sessions could have produced detectable significant changes. Furthermore, some tests normally require a 3-month intervention period in order to measure any change which is the case for the SCQ. Perhaps at least twelve sessions each week would probably give enough time to get better measurable changes. These are just speculations, however, studies in the future should consider including larger data sets to analyze a linear relationship between dosage and DMP impact predictor.

The tools used in this study were reportedly identified with satisfactory reliability, validity and sensitivity to capture the intervention effects (Mieloo et al., 2012; Avcil et al., 2015; Stone et al., 2015). However, the questionnaires used required parent and teacher perspectives on the children’s wellbeing and thus, do not capture the children’s perspectives of their own wellbeing. Such measures of wellbeing are often difficult to administer to children, particularly those lacking the appropriate language skills. Currently, there are escalating arguments on who speaks for children with ASD and empowering children’s voice on their wellbeing by recognizing their perspectives in intervention-based research (Devlin and Appleby, 2010; Moula et al., 2020). The belief here is that data from first-person opinion can provide trustworthy and rich information (Kellett, 2011). Therefore, qualitative and arts-based measures were used to bypass the language abilities of the children but restricted the quantitative data to teachers’ and parents’ perspectives only. This is not unusual in children’s studies. Among the studies included in the systematic review (Aithal, 2020), only one study (Samaritter, 2015) employed a self-reporting method i.e., the Youth Self Report (Achenbach et al., 2008). This study, however, involved adolescent participants with competent language abilities.

With regards to execution of the research design, this study included randomization and allocation concealment. However, it did not include blinding to reduce placebo in the measurements using rating scales answered by teachers and parents was impractical. Nonetheless, there are possibilities to achieve this by implementing clinician administered observational tools with assessor blinding measures where the clinicians administering the tests are not aware which group of children is receiving the intervention and which group is not. The other option to reduce the bias and placebo in testing would be randomized retrospective video analysis where the analyst is blinded about the participants’ intervention.

The debate over ASD as a disorder and neurodiversity movement arguing the condition as distinct cognitive style and not a disorder reflect on the inconsistencies in terminologies used and also the differences in strengths and deficit models (Miller et al., 2016). With these differences in mind, although the intervention aimed at resource enhancement and adopted strength-based model, appropriate tools were not available to measure the increment of strengths with a positive attitude (Mottron, 2017). Despite the neurodiversity movement, available validated tools are mainly based on deficit models, mostly focusing on ASD’s negative symptom reduction or decrement in challenges. To make a complete shift to a strength-based approach, the development of tools which capture improvements to match the intervention principles are necessary. Furthermore, tools which capture the embodied components would also be valuable to capture the nuances of DMP. Tools which are malleable through intervention might be able to provide more meaningful outcomes and thus highlights future targets of research.

The study adopted a crossover design to pilot an early stage trial. Since this study aimed to explore outcome measures related to ASD i.e., SCQ and SDQ in children with ASD, crossover design was applicable due ASD’s long-term or pervasive nature where there is no immediate cure. One of the strengths of this design is that it allows for greater homogeneity in the sample as it eliminates between-subject variability (Maclure, 1991). The design can also provide two folds of the actual sample size and more importantly from an ethical point of view all the participants receive intervention at some point of time and do not miss out the opportunity to participate on a random chance. However, some researchers have argued that even when crossover trials are properly implemented with a washout period, there is less clarity in addressing the impact of a carryover effect (Senn et al., 2004). This design also restricts the scope for symmetrical follow-up data collection as in the parallel group research design with separate experimental and control groups. Therefore, for exploring long lasting effects of the intervention traditional RCTs are highly recommended.

Intention to treat (ITT) analysis was used in the current study since this approach tends to avoid various misleading findings that can arise in intervention research. ITT analysis includes every subject who is randomized. A key advantage of this approach is that it estimates the efficacy of the intervention and is more accurate as it accepts that non-compliance and protocol deviations are likely to occur in clinical practice (Gupta, 2011). In the current study, rates for session attendance were reasonably good with only three out of the included 26 participants not completing the intervention, and 23 of the participants partaking in 70% or more of the sessions. The outcome measures, which were completed by either parents or teachers were, however, collected for all participants, irrespective of whether the child completed the intervention. This allowed for the ITT approach which is difficult to achieve when outcome data is missing (Altman, 2009; Can et al., 2011; Bell et al., 2014). In line with Dziura et al. (2013) strategies for the prevention of missing data is key to minimizing the problem of missing data and highlights an advantage for conducting the study in a school setting i.e., as a result there was negligible amount of missing data. Teachers were accessible within schools to complete questionnaires and the data from the parents were collected during their termly school visits. This made data acquisition fairly straightforward.

## Conclusion

Findings from the current study show promising effects of the DMP intervention on measures of children’s wellbeing including measures specific to ASD. Modest improvements in social communication scores and in strengths and difficulties suggest that, group DMP has been effective. A crossover design was employed in the current study and although no carryover or period effects were found, it is recommended to employ a different study design in the future that will allow for follow-up measures to be conducted to assess the long-term effects of the DMP intervention. Future studies should also consider the “dosage” period for the DMP intervention, utilizing a longer intervention period in order to detect intervention effects and enhance intervention related improvements, especially for those children who are deemed to be more in need of receiving support for their wellbeing. Neuroimaging studies are needed to better understand the neural mechanisms contributing to the enhanced change in SCQ and SDQ scores as a result of the DMP intervention. This is particularly poignant given that the current study shows changes, particularly those in relation to social communication, in a group of children with a diagnosed neurodevelopmental disorder. The findings of the study indicate that social, communication and emotional independence can be improved through an intervention that utilises the creative arts, highlighting the value of these creative therapies for improving the lives of young vulnerable groups who typically, due to a lack of verbal skills, would be unsuited to more traditional talking therapies. Future studies could include large scale multi-centered RCT to empirically validate the effectiveness of DMP for children with ASD. Studies of this type may enable the intervention to be more readily available for children with ASD and can potentially have long-term impact on improving productivity, reducing economic burden and overall wellbeing in the society.

## Data Availability Statement

The raw data supporting the conclusions of this article will be made available by the authors, without undue reservation.

## Ethics Statement

The study received ethical approval by the Research Ethics committee of the Faculty of Arts and Sciences, Edge Hill University. Written informed consent to participate in this study was provided by the participants’ legal guardian/next of kin.

## Author Contributions

SA was responsible for designing, executing, analyzing and writing up the current manuscript. It was conducted as a part of her doctoral thesis. VK guided the development process of the study and provided corrections for the manuscript as the director of studies for SA’s doctoral thesis. She also acted as the clinical supervisor for the DMP intervention programme. SM and TK are the members of the supervisory team. They contributed to the development of study, revisions and edits of the manuscript. JP as the member of the supervisory team took the lead role in the statistical analysis, guided the writing-up process and also contributed to the editing of the manuscript.

## Conflict of Interest

The authors declare that the research was conducted in the absence of any commercial or financial relationships that could be construed as a potential conflict of interest.
